# Tumorigenesis and Tumor Microenvironment in Lung Cancer

**DOI:** 10.3390/cimb48030247

**Published:** 2026-02-26

**Authors:** Puneet Dhillon, Moshe Carroll, Haiying Cheng

**Affiliations:** Department of Oncology, Albert Einstein College of Medicine, Montefiore Einstein Comprehensive Cancer Center, Bronx, New York, NY 10461, USA; pudhillon@montefiore.org (P.D.); moshe.carroll@einsteinmed.edu (M.C.)

**Keywords:** lung cancer, tumor microenvironment, cancer-associated fibroblasts, immunotherapy, metastasis, brain metastasis, TGF-β, IL-6, perioperative therapy

## Abstract

Lung cancer remains a leading cause of cancer mortality worldwide and continues to impose substantial clinical and economic burdens. Beyond tumor-intrinsic oncogenic drivers, disease progression and therapy response are shaped by the tumor microenvironment (TME), including immune cells, cancer-associated fibroblasts (CAFs), endothelial cells, extracellular matrix, inflammatory mediators, etc. In lung cancer, chronic injury from tobacco smoke, airway disease, and treatment itself remodels local tissue programs that can either support antitumor immunity or promote immune exclusion, fibrosis, and metastatic seeding. Here, we analyze recent evidence linking lung tumorigenesis to TME ecology across histologies, with emphasis on CAF heterogeneity, spatial organization of immune niches, and the distinct microenvironments that govern organ-specific metastasis (including brain metastasis). We also evaluate emerging therapeutic strategies that aim to target or reprogram the TME, including perioperative immune checkpoint blockade, combined immunotherapy–radiotherapy approaches, and pathways such as IL-6 and TGF-β that coordinate immune suppression and stromal remodeling. Finally, we outline key gaps and potential future directions, such as longitudinal and spatial multi-omics, better biomarkers of stromal state, and trial designs that account for dynamic microenvironmental adaptation.

## 1. Introduction

Despite advances in screening and therapy, lung cancer remains a major contributor to cancer mortality, and overall outcomes are still poor for patients diagnosed with locally advanced or metastatic disease [1,2,3]. Population-based estimates highlight both the magnitude of the problem and the persistence of stage-dependent survival gaps, underscoring the need for prevention, earlier detection, and more durable systemic control [4]. Beyond clinical impact, lung cancer is a substantial driver of cancer-related healthcare spending; forecasts of national cancer-care costs indicate that demographic changes alone can produce large increases in expenditures over time [1,2].

Non-small-cell lung cancer (NSCLC) accounts for the majority of lung cancers and mainly encompasses adenocarcinoma and squamous cell carcinoma, with treatment increasingly shaped by molecular targeting and immune checkpoint blockade [5,6]. Small-cell lung cancer (SCLC) remains a highly aggressive neuroendocrine malignancy; while it has been historically treated as a single entity, recent work supports biologically distinct subtypes defined by lineage transcription factors and inflammatory programs, with potential therapeutic implications [7]. Across histologies, tumor progression should be viewed not only through the lens of tumor genetics but also through the ecological context in which tumor cells evolve [7,8]. Overall treatment efficacy in lung cancer remains limited by drug resistance, mechanisms of which are classified as intrinsic or acquired, both of which often coexist [8]. Intrinsic resistance is mediated by tumor heterogenicity, established genetic mutations, and intrinsic host defenses against drug toxicities, whereas acquired resistance mechanisms are characterized by the activation of secondary oncogenes, mutations in drug targets, or changes in the tumor microenvironment (TME) [8,9,10].

The TME comprises immune and stromal components that can restrain tumor growth or, conversely, support immune evasion, therapy resistance, and metastatic spread [9,10,11]. Conceptual frameworks developed across cancer types emphasize that the microenvironment is not a passive bystander but an active participant in tumor initiation and outgrowth [10,11]. In lung cancer, the baseline immune landscape of the airway and alveolus, environmental exposures, and comorbid inflammatory lung disease together establish a tissue context that can bias the TME toward immune surveillance or chronic, tumor-promoting inflammation. In this review, we describe the various components of the TME in NSCLC and its contributions to metastasis and to primary lung cancers and secondary lung metastases. We also discuss current therapeutic agents and their relationship to the TME, as well as future therapies and developments in the context of the TME in lung cancer. The goal of this review is to serve as a concise synthesis of lung tumor microenvironment biology, integrating oncogenic signaling, immune modulation, and therapeutic strategies into a framework accessible to a board audience, including clinicians and researchers.

## 2. Foundations of the Lung TME

The TME is a complex and dynamic network comprising numerous cell types and signaling pathways including cancer-associated fibroblasts (CAFs), endothelial and immune cells, and signaling molecules, as well as the surrounding vasculature and extracellular matrix [9,10,11,12,13,14,15]. In the lungs, extracellular matrix (ECM) cells may include various structural cells and their products and immune cells, including resident alveolar macrophages and dendritic cells. Cytokines such as TGF-β, IL-6, and IL-10 promote immune suppression, epithelial–mesenchymal transition, and tumor growth, while pro-inflammatory cytokines can also drive chronic inflammation that supports oncogenesis [12,13]. Chemokines including CCL2, CXCL8, and CXCL12 orchestrate the recruitment and spatial organization of immune and stromal cells, often favoring the accumulation of immunosuppressive myeloid populations [14,15]. Bone-marrow-derived cells—such as monocytes, myeloid-derived suppressor cells (MDSCs), and neutrophils—are mobilized by these signals and home to the tumor site, where they differentiate into tumor-associated macrophages or other pro-tumorigenic phenotypes. The population of tissue-resident memory T cells resides in the lung airways and tumor-associated macrophages (TAMs). Alterations in the TME influence carcinogenesis, therapy selection, therapy response, and resistance to therapy [15].

The lung is continuously exposed to inhaled antigens and therefore maintains tightly regulated immune surveillance. Resident macrophages, dendritic cells, innate lymphoid populations, and tissue-resident memory T cells coordinate barrier defense while limiting immunopathology [12]. Tumorigenesis can hijack these homeostatic programs: myeloid skewing, dysfunctional antigen presentation, T-cell exhaustion, and altered chemokine gradients can progressively shift the lung TME toward immune tolerance [13,14,15,16,17,18]. These changes occur alongside stromal remodeling (such as angiogenesis, extracellular matrix deposition, and fibroblast activation) that can physically and biochemically restrict immune infiltration [13]. Variations in the TME have also been studied as prognostic and predictive indicators for outcomes and survival. For instance, studies have found that presences of lymphocytes, particularly CD8+ T cells and M1-macrophages, predict longer survival [16].

CAFs are a particularly highly heterogeneous and therapeutically relevant stromal population. The primary functional CAF subsets include myofibroblast (myCAFs), antigen-presenting (apCAFs), and inflammatory (iCAFs). First, myCAFs are primarily associated with ECM synthesis and deposition [17]. These cells express elevated levels of α-smooth muscle actin (α-SMA), have contractile properties, and have strong responsiveness to TGF-β, driving myCAF differentiation and, thus, ECM accumulation. Furthermore, myCAFs are [18,19] typically positioned in close proximity to tumor cells. This spatial relationship is thought to provide a dense ECM environment that effectively shields the tumor from treatment. However, the exact role of myCAFs in tumor growth is complex. Although a high proportion of myCAFs correlates with increased ECM deposition and poorer prognosis, other studies demonstrate that myCAFs can restrain tumor progression, as their depletion and loss of ECM collagen production accelerate growth in certain cancers [20,21,22,23,24]. Consequently, myCAFs may exhibit dual functions relating to tumor growth.

Second, apCAFs likely originate from mesothelial cells after differentiation induced by IL-1 and TGF-β [25]. These cells are distinguishable by MHC-II and CD74 expression, activation by IFN-γ and STAT1, and their ability to present antigens to CD4+ T cells [26]. However, apCAFs lack the necessary expression of costimulatory molecules and therefore cannot activate immune cells. Indeed, apCAFs have been shown to induce the transformation of naive CD4+ into regulatory T-cells. Thus, apCAFs are largely hypothesized to be immunosuppressive, particularly in breast and pancreatic tumors [27,28,29]. However, a recent study has shown that, in NSCLC, apCAFs actively promote CD4+ T cells through MHC-II and T-cell receptor binding as well as production of C1q, which is protective from apoptosis [30]. Another recent study on gastric cancer tumors demonstrated that apCAFs are preferentially located near tertiary lymphoid structures and not only promote T cell activation and cytotoxicity but also engage in a feedback loop with pro-inflammatory macrophages to bolster immune responses [31]. Thus, the function of apCAFs may be context-dependent and vary depending on the specific tumor microenvironment.

Third, iCAFs are characterized by low α-SMA, high IL-6 expression, and a proinflammatory phenotype [32]. Induced by tumor-derived IL-1 through the LIF/JAK/STAT pathway, they are typically located distant from tumor cells and secrete a broad array of cytokines and chemokines, including IL-6, IL-11, CXCL1/2, LIF, IL-8, CXCL12, HGF, CCL17, and HAS1/HAS2, which drive hyaluronic acid synthesis [19,26,32,33]. iCAFs promote tumor progression by secreting cytokines via JAK/STAT and NF-κB to recruit immunosuppressive MDSCs, M2 macrophages, and Tregs and by releasing CXCL12 to drive immune evasion and treatment resistance [34,35,36]. Additionally, iCAFs and myCAFs exhibit plasticity, interconverting between phenotypes in response to IL-1 or TGF-β, favoring iCAFs or myCAFs, respectively [26]. Spatially, iCAFs often reside outside the ECM constructed by myCAFs, an arrangement that may facilitate the efficient dissemination of cytokines throughout the TME. Furthermore, iCAF-derived factors play diverse tumorigenesis roles [37]. For example, CXCL1 drives neoangiogenesis, EMT, and cancer stem cell expansion; CXCL12 and CCL17 further support immunosuppression; and IL-6 exerts systemic effects, including suppression of hepatic ketogenesis, elevated glucocorticoids, and contribution to immunotherapy resistance [37,38,39,40]. Consequently, an elevated presence of iCAFs in the TME is strongly associated with poor prognosis and treatment resistance across multiple cancer types [41].

**Figure 1:**
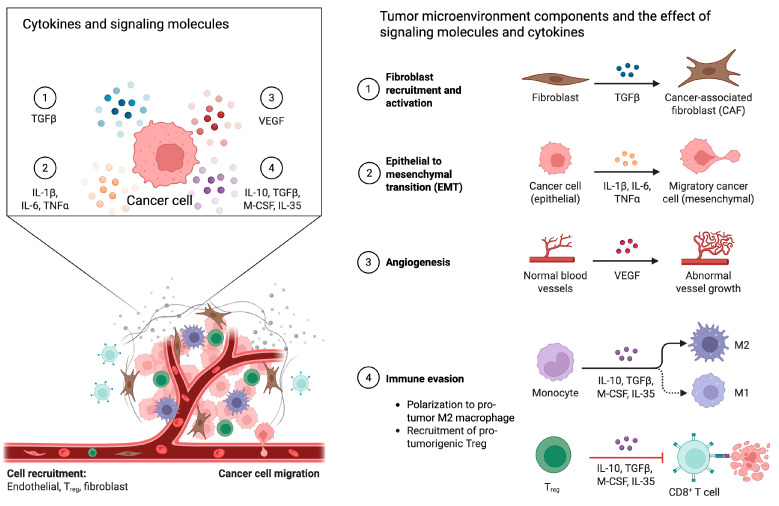
Components of the tumor microenvironment. This schematic view shows the key interactions between cancer cells and cytokines and signaling molecules (**left**) and the resulting effects (**right**). Created in BioRender. Dhillon, P. (2026) https://BioRender.com/vqf74ew.

In NSCLC, specific CAFs are shown to play significant roles in promoting tumor cell survival, immune suppression, and resistance to targeted therapies through paracrine signaling and extracellular matrix remodeling [42,43]. For example, podoplanin-positive CAFs have been linked to primary resistance to EGFR tyrosine kinase inhibitors in lung adenocarcinoma [44], and CAF-derived secreted factors (including IGF-binding proteins) can modulate drug sensitivity in a context-dependent manner [45]. Recent mechanistic work further supports that CAF programs can actively seed metastatic competence: a circRNA-driven pathway (circNOX4) was shown to activate an inflammatory CAF niche via a FAP/IL-6 axis and to promote metastatic colonization in vivo [46]. Figure 1 shows a schematic view of key components of the tumor microenvironment.

Recent advances in spatial transcriptomics and high-resolution imaging show that spatial heterogeneity, or distinct immune-cell niches, and cross-talk between these regions exist in the TME [42,43,44,45]. These niches are typically described as immune-rich, or areas where there exist a variety and high density of immune cells and which have anti-tumor activity, and immune-poor, or areas where tumor growth may be promoted through change in stiffness, hypoxia, or immune cell behavior [43]. Spatially resolved profiling has begun to clarify how stromal and immune states are organized in situ. Rather than being uniformly mixed, lung tumors often contain spatially segregated immune niches, ranging from T cell-rich regions with tertiary lymphoid structures to immune-excluded areas gated by dense stroma and suppressive myeloid cells. Such spatial architectures are likely to influence response to immunotherapy and may explain why bulk ‘immune hot’ versus ‘cold’ classifications fail to capture key intratumoral variability [11,44].

Tobacco smoke and chronic lung disease, such as chronic obstructive pulmonary disease (COPD), add an additional layer of complexity by altering both epithelial injury-repair programs and immune tone. Smoking and COPD are associated with persistent inflammation, macrophage and neutrophil activation, and fibrotic remodeling, all of which can reshape the pre-tumor niche [9,11,44,45,46]. Importantly, these exposures may have bidirectional effects on therapy: higher mutational burden can increase neoantigen load, while chronic inflammation and stromal remodeling can foster immune suppression and impaired T-cell trafficking. Future work should explicitly model these comorbidity-driven states when interpreting TME biomarkers [3,47].

## 3. TME and Metastatic Ecology

Metastasis is not solely a property of disseminating tumor cells; it depends on a receptive ‘soil’ shaped by the resident tissue microenvironment [48]. The lung is both a frequent source of metastatic spread and a common site of colonization for other solid tumors [49]. In primary lung cancer, local immune and stromal programs can facilitate early dissemination, and therapy-induced remodeling may further reshape the metastatic niche [50]. Understanding which TME states predict dissemination, and whether they are reversible, remains a critical translational challenge.

Metastasis effects of inflammation include ubiquitin–CXCR4 signaling axis, elevated levels of E-selectin expression in lung tissue, NF-κB activation in airway epithelial cells, increased CCL2 expression, and macrophage infiltration in the lung microenvironment. Signaling pathways play an important role and Nuclear Factor Kappa B (NF-κB) and Signal Transducer and Activator of Transcription 3 (STAT3) pathways contribute to chronic inflammation, immune evasion, and angiogenesis. CCL2 promotes the differentiation of monocytes into metastasis-associated macrophages and the formation of pre-metastatic niche, accelerating the colonization and growth of metastatic tumor cells [27]. Induction of macrophage M2 polarization secretes CXCL12 and promotes metastatic niche formation through the CXCL12/CXCR4/NF-κB signaling pathway. The premetastatic niche (PMN) describes a microenvironment in organs without metastasis that foments tumor seeding and metastasis. It primes remote locations for the eventual infiltration of disseminated tumor cells (DTCs). After the arrival at metastatic sites, disseminating tumor cells could be eliminated by immune cells or enter a state of dormancy by exiting proliferative cell cycle [50,51]. Some lesions can therefore occur years later.

Brain metastases represent a major clinical inflection point in NSCLC and arise in a distinct immune–stromal context shaped by the blood–brain barrier, microglia, astrocytes, and specialized vascular niches. In brain metastasis, T cell infiltration is evident in BM, with higher densities of CD8+ and CD45RO+ T cells correlating with improved prognoses. Immune composition (reduced TILs/PD-L1), BBB remodeling, microglia (M2), astrocytes, and STAT3/PI3K-AKT-mTOR signaling are all directly relevant to immunotherapy sensitivity [51,52]. Intracranial lesions often display reduced T-cell infiltration and altered myeloid programs compared with extracranial sites, which can contribute to treatment resistance [51,52]. These biological constraints help explain why systemic therapies with proven extracranial activity do not uniformly translate to intracranial control.

Clinical strategies that integrate immunotherapy with modalities that alter local tissue ecology, most notably radiotherapy, aim to overcome these barriers. Prospective evidence also supports activity of chemo-immunotherapy in untreated brain metastases: in a phase II study (ATEZO-BRAIN), atezolizumab combined with carboplatin and pemetrexed demonstrated intracranial activity and an acceptable safety profile in patients with advanced nonsquamous NSCLC with untreated brain metastases [53,54]. While encouraging, these results also highlight the need for mechanistic correlative studies to define which brain–TME states are associated with durable intracranial responses.

## 4. Therapies That Target or Remodel the TME (Early and Metastatic Settings)

Therapies that target tumor cells inevitably perturb the surrounding microenvironment [55]. In lung cancer, this is increasingly intentional: perioperative immune checkpoint blockade aims to eradicate micrometastatic disease, and combination strategies in advanced disease attempt to convert immune-suppressed or immune-excluded tumors into inflamed, therapy-responsive states [55,56,57,58].

Immune checkpoint inhibitors (ICIs) represent the first wave of therapies that directly reprogram the TME, shifting it from an immunosuppressive niche toward effective antitumor immunity. In lung cancer, this foundation has enabled a therapeutic ladder that extends from targeting TME components, such as tumor-associated macrophages (TAMs), CAFs, and the ECM, to perioperative ICI strategies integrated with surgery [56,57]. ICIs targeting PD-1/PD-L1 are approved across metastatic, adjuvant, and neoadjuvant settings in resectable non-small-cell lung cancer, including neoadjuvant chemo-immunotherapy and perioperative ICI approaches that improve pathologic response and event-free survival [55,56,57,58].

Perioperative immunotherapy has an established proof-of-principle that microenvironmental reprogramming can translate into improved clinical outcomes. In the phase III CheckMate 816 trial, neoadjuvant nivolumab plus chemotherapy improved event-free survival, overall survival, and increased pathological complete response rates relative to chemotherapy alone, without compromising the feasibility of surgery [59,60]. Similar perioperative approaches with pembrolizumab (KEYNOTE-671) and durvalumab (AEGEAN) have reported event-free survival benefits [61,62], and adjuvant atezolizumab improved disease-free survival after resection and chemotherapy in IMpower010 [63]. These data support the perioperative window as an opportunity to interrogate therapy-induced TME changes and to refine biomarkers beyond baseline PD-L1 [59,60,61,62,63].

Nevertheless, perioperative regimens introduce new practical and biological questions. Treatment-related inflammation and fibrosis can complicate pathologic assessment and may pose technical challenges during resection; standardized reporting of surgical endpoints and tissue quality is therefore essential [56]. Biologically, early exposure to immune checkpoint blockade may also select for immune escape programs (e.g., antigen presentation defects or interferon pathway adaptations) that are not captured by short-term radiographic response [58,59]. Longitudinal sampling and harmonized correlative endpoints are needed to distinguish productive immune priming from transient inflammation [59,60,61,62,63].

In metastatic disease, attempts to remodel the TME include targeting inflammatory cytokine axes and stromal pathways. IL-6 signaling is one candidate node, given its links to cachexia, systemic inflammation, and myeloid skewing [64]. An observational study of tocilizumab in advanced NSCLC with IL-6-elevated cachexia suggested feasibility and generated hypotheses for prospective testing [65,66,67]. In contrast, broad enthusiasm for TGF-β pathway inhibition has been tempered by the context-dependent biology of TGF-β and mixed clinical results; careful patient selection and biomarker-driven combinations are likely prerequisites for success [42].

Targeting TGF-β to relieve immune exclusion is a major area of investigation [68,69,70,71,72]. Bintrafusp alfa, a bifunctional fusion protein targeting PD-L1 and sequestering TGF-β, showed early signals of activity, but a phase III comparison against pembrolizumab in PD-L1-high NSCLC did not demonstrate superior efficacy [73]. Reviews and mechanistic studies continue to support TGF-β as a rational target, particularly in stroma-rich and immune-excluded tumors, but also emphasize the risk of on-target toxicities and the need to match inhibitor class to disease context [74].

STAT3, a key transcription factor in lung cancer, drives tumor growth and immune suppression via the mTOR and JAK pathways. Activation of STAT3 is thought to play an important role in tumor resistance to conventional and targeted small-molecule therapy, especially for mutations in the JAK/STAT pathway [58]. Inhibiting STAT3 pathways may slow cancer progression. Promising results have been observed with mTOR inhibitors like CC-115 and Vistusertib, especially when combined with immune checkpoint inhibitors, and with JAK inhibitors such as Ruxolitinib, AZD4205, and Filgotinib, canakinumab anti-ILB [50,75,76]. Another possible strategy targeting of the carcinogenic effects of CAFs and fibroblast activation protein (FAP)-positive CAFs. Unfortunately, clinical studies that employ FAP monoclonal antibodies or enzyme inhibitors have largely not been found to yield significant benefits, potentially due to insufficient drug-dependent cell cytolysis [13,64,75]. However, a recent case report of two patients with FGFR-mutant NSCLC found that pemigatinib, an FGFR2 inhibitor, had prolonged clinical benefit [77]. Thus, continued research is necessary to better elucidate the potential benefits of FAP/FGFR inhibition [13].

## 5. Clinical Translation and Biomarker Considerations

The clinical relevance of lung tumor microenvironment biology is ultimately judged by its ability to refine patient selection, guide combination strategies, and inform surveillance for recurrence. Perioperative trials provide a uniquely tractable platform because they pair a defined treatment window with access to pretreatment tissue, treatment-induced changes, and resection specimens [59,60]. The improvements in event-free survival and pathologic response reported with perioperative PD-1/PD-L1 blockade plus chemotherapy support the concept that early immune priming can translate into durable control [59,60,61,62,63]. However, the magnitude and durability of benefit likely depend on baseline immune architecture, stromal exclusion programs, and suppressive myeloid states that are incompletely captured by PD-L1 alone. PD-L1 and other markers such as tumor mutational burden are incomplete biomarkers due to biologic heterogeneity, spatial dependence, and technical variability and may not capture the whole TME. There are implementable approaches that are promising such as standardized PD-L1 with multiplex IHC/IF panels, including Imaging Mass Cytometry (IMC), Multiplexed Ion Beam Imaging (MIBI), and Cyclic Immunofluorescence (CycIF), spatial immune-architecture features (e.g., immune exclusion vs tertiary lymphoid structure metrics), and stromal-state signatures that may guide rational combinations [78,79]. These methods are not clinically standard at this time though may hold significant significance. For instance, Moutafi et al. identified and validated CD66b as a biomarker indicative of resistance to ICI treatment in NSCLC patients using digital spatial profiling/IF, though multi-institutional studies and clinical trials are imperative in this space [80].

To strengthen mechanistic inference and biomarker credibility, correlative endpoints should be pre-specified and matched to the biological question. Some potential priorities include: (i) spatially resolved assessment of immune context (e.g., immune exclusion versus tertiary lymphoid structure features) rather than cell-type abundance alone; (ii) parallel measurement of tumor-intrinsic states and stromal programs to avoid attributing response solely to immunity; (iii) standardized sampling and reporting across sites and time points to enable cross-trial comparisons. These principles are particularly important when interpreting fibroblast and myeloid signatures, where marker-based definitions do not always correspond to stable functional programs.

In metastatic disease, organ-specific microenvironments can decouple systemic response from site-specific control, and the brain is an instructive example. Prospective studies have demonstrated intracranial activity of chemo-immunotherapy in untreated brain metastases, but durable intracranial control is likely to require better definitions of the intracranial niche and mechanisms of resistance [53]. Whenever feasible, biomarker strategies should incorporate matched sampling of extracranial and intracranial lesions (or surrogates) alongside intracranial endpoints. Selected clinically relevant TME-modulating strategies and their supporting evidence are summarized in Table 1.

## 6. Conclusions

In conclusion, the lung TME has been established as an ecosystem that critically shapes tumor initiation, progression, immune evasion, and therapeutic response and engages in interactions with cancer cells that influence disease trajectory and treatment outcomes. Though the role of the TME is well-recognized, there is much work that needs to be done in terms of translating patient therapeutics and recognizing resistance. Ultimately, a comprehensive and clinically informed understanding of the TME will be central to advancing precision medicine approaches and improving outcomes for patients with lung cancer. A persistent limitation in lung TME research is that most mechanistic insights derive from cross-sectional sampling. Yet the microenvironment is dynamic—changing with smoking cessation, infection, treatment [67], and metastatic progression. Prospective, longitudinal sampling (including minimally invasive approaches such as liquid biopsies and serial imaging) is essential to link TME state transitions to clinical events such as recurrence and organ-specific progression [11].

Some potential steps could accelerate translation, for example, (i) incorporation of spatial and multi-omic profiling into trials, with pre-specified hypotheses about immune infiltration and stromal remodeling, and (ii) metastasis-focused correlative studies, particularly for brain metastases, that profile both lesions and matched extracranial sites to define mechanisms of intracranial resistance. Table 2 includes selected ongoing clinical trials that implement targets in the TME [68,69,70,71,72,74]. Together, these steps would help move the field from descriptive atlases toward actionable microenvironmental targets and biomarker-driven trial design.

## Figures and Tables

**Table 1:** Selected therapies with evidence of TME or metastatic-ecology impact in lung cancer.

Therapy	Disease Stage	Key Evidence (PMID/Trial)	TME/Niche Rationale
Nivolumab + platinum-doublet chemotherapy (PD-1 blockade)	Neoadjuvant (resectable NSCLC)	CheckMate 816 (PMID: 35403841)	Augments antitumor T-cell priming in presence of intact tumor antigen; may increase inflamed niches.
Pembrolizumab + chemotherapy → adjuvant pembrolizumab	Perioperative (resectable NSCLC)	KEYNOTE-671 (PMID: 37272513)	Perioperative priming and maintenance; tests durability of immune reprogramming.
Durvalumab + chemotherapy (perioperative)	Perioperative (resectable NSCLC)	AEGEAN (PMID: 37870974)	Combines cytotoxic debulking with PD-L1 blockade; potential effects on myeloid and stromal states.
Atezolizumab (PD-L1 blockade)	Adjuvant (resected NSCLC after chemo)	IMpower010 (PMID: 34555333)	Sustains antitumor immunity post-resection; aims to clear micrometastatic disease.
Atezolizumab + carboplatin + pemetrexed	Metastatic NSCLC with untreated brain metastases	ATEZO-BRAIN (PMID: 37603816)	Systemic immunotherapy in the setting of brain immune constraints; intracranial activity suggests partial niche penetration.
Tocilizumab (IL-6R blockade)	Advanced NSCLC with inflammatory cachexia	Observational study (PMID: 39523982)	Targets IL-6-driven systemic inflammation; may reduce myeloid skewing and cachexia-associated immune suppression.
Bintrafusp alfa (PD-L1 + TGF-β trap)	Advanced NSCLC (PD-L1-high, 1L)	Phase 3 vs pembrolizumab (PMID: 37597750)	Attempts to relieve TGF-β-mediated immune exclusion while blocking PD-L1; efficacy not superior in unselected PD-L1-high population.
circNOX4/FAP/IL-6 axis (CAF niche)	Preclinical/translational	Mechanistic study (PMID: 38459511)	Illustrates how inflammatory CAF programs can drive metastasis; highlights CAF niche as actionable component.

**Table 2:** Selection of clinical trials and therapeutics and targeted components of the TME.

Trial	Therapy	Target Component of TME
Be6A Lung-01/Be6A Lung-02 (2025)	Sigvotatug vedotin (±pembrolizumab)	Integrin αvβ6 on tumor/stromal interface (TME adhesion/ECM)
AK104-202 (Ongoing; 2024–2025)	AK104 cadonilimab (bispecific anti-PD-1/CTLA-4)	Immune checkpoint inhibition to modulate T cell activity in TME
KN046-202 (Phase 2)	KN046 (bispecific anti-PD-L1/CTLA-4)	Immune checkpoints > T cell activation and tumor immunity
NCT04931654 (Phase 1)	AZD7789 (anti-PD-1/TIM-3 bispecific)	Dual immune checkpoint blockade to overcome TME immunosuppression
NCT02665416 (Phase 1)	Selicrelumab + Vanucizumab	CD40 agonist (immune activation) + ANGPT2/VEGF (angiogenesis/vasculature)
NCT02857920 (Phase 1/2)	NK cell immunotherapy + Bevacizumab	NK cells + anti-angiogenic targeting of VEGF-A
NCT02923739 (Phase 2)	Emactuzumab (anti-CSF-1R) + Bevacizumab + Paclitaxel	Tumor-associated macrophages (CSF-1R) + angiogenesis (VEGF-A)
NCT02760797 (Phase Ib)	Emactuzumab + Selicrelumab (TAM modulation + immune activation)	Tumor-associated macrophages (CSF-1R) + CD40 on APCs

## Data Availability

No new data were created in this review.
